# Identification of EGFR as a Novel Key Gene in Clear Cell Renal Cell Carcinoma (ccRCC) through Bioinformatics Analysis and Meta-Analysis

**DOI:** 10.1155/2019/6480865

**Published:** 2019-02-13

**Authors:** Sheng Wang, Zhi-hong Yu, Ke-qun Chai

**Affiliations:** ^1^The Second Clinical Medical College, Zhejiang Chinese Medicine University Hangzhou, Zhejiang 310053, China; ^2^Department of Oncology, Tongde Hospital of Zhejiang, Hangzhou, Zhejiang 310012, China

## Abstract

Clear cell renal cell carcinoma (ccRCC) was the most aggressive histological type of renal cell carcinoma (RCC) and accounted for 70–80% of cases of all RCC. The aim of this study was to identify the potential biomarker in ccRCC and explore their underlying mechanisms. Four profile datasets were downloaded from the GEO database to identify DEGs. GO and KEGG analysis of DEGs were performed by DAVID. A protein–protein interaction (PPI) network was constructed to predict hub genes. The hub gene expression within ccRCC across multiple datasets and the overall survival analysis were investigated utilizing the Oncomine Platform and UALCAN dataset, separately. A meta-analysis was performed to explore the relationship between the hub genes: EGFR and ccRCC. 127 DEGs (55 upregulated genes and 72 downregulated genes) were identified from four profile datasets. Integrating the result from PPI network, Oncomine Platform, and survival analysis, EGFR, FLT1, and EDN1 were screened as key factors in the prognosis of ccRCC. GO and KEGG analysis revealed that 127 DEGs were mainly enriched in 21 terms and 4 pathways. The meta-analysis showed that there was a significant difference of EGFR expression between ccRCC tissues and normal tissues, and the expression of EGFR in patients with metastasis was higher. This study identified 3 importance genes (EGFR, FLT1, and EDN1) in ccRCC, and EGFR may be a potential prognostic biomarker and novel therapeutic target for ccRCC, especially patients with metastasis.

## 1. Introduction

Kidney cancer, one of the most common malignant tumor globally, was estimated where nearly 64,000 new cases in the USA were diagnosed in 2017 [1] and rose by 2–4% each year steadily [2]. Clear cell renal cell carcinoma (ccRCC) was the most aggressive histological type of renal cell carcinoma (RCC) and accounted for 70–80% of cases of all RCC [3, 4]. Although the 5-year survival of ccRCC patients with early and localized disease was more than 90%, for patients with distant metastasis, the 5-year survival drops to 12% [5], and almost 20-40% patients would experience distant metastasis [6]. Due to resistance to standard chemotherapy and radiotherapy, ccRCC patients with metastatic had worse prognosis [6]. Hence, it is essential to identify the underlying molecular mechanisms of ccRCC, which may be conducive to the risk assessment of disease and guide clinical decision-making and develop novel diagnostic and therapeutic strategies for ccRCC.

The molecular pathogenesis of carcinoma was complex, which was associated with inactivation and mutation of tumor suppressor genes and activation of oncogene [7]. Recently, bioinformatics analysis based on gene expression microarrays has emerged as an efficacious novel approach to identify new genes and comprehend the underlying molecular mechanisms of cancer [8]. For instance, Wang et al. [9] have reported that RFC5, significantly overexpressed in lung cancer, was closely related to the prognosis of lung cancer and might be a potential biomarker and therapeutic target for lung cancer. In addition, Li et al. [10] have identified 451 DEGs between triple negative breast cancer (TNBC) and normal breast tissues and ten hub genes like CCNB1 may be key prognostic factor and potential target for TNBC therapy.

In the current study, four gene chips (GDS505, GDS507 [11], GDS2880, and GDS2881 [12]) were downloaded from NCBI-Gene Expression Omnibus (GEO) database (https://www.ncbi.nlm.nih.gov/geo/) to detect the differentially expressed genes (DEGs) between ccRCC tissue and normal renal tissue. Kyoto Encyclopedia of Genes and Genomes (KEGG) pathway enrichment analysis and Gene Ontology (GO) functional annotation analysis were applied. Then a protein–protein interaction (PPI) network was performed to identify hub genes associated with ccRCC. After screening and confirmed by Oncomine dataset (https://www.oncomine.org) and UALCAN (http://ualcan.path.uab.edu), epidermal growth factor receptor (EFGR) was deemed to a key factor and potential target for the treatment of ccRCC. In order to further explore the relationship between EGFR and ccRCC, we performed a meta-analysis.

## 2. Materials and Methods

### 2.1. Bioinformatics Analysis

#### 2.1.1. Microarray Data

Four profile datasets (GDS505, GDS507, GDS2880, and GDS2881) were downloaded from the GEO database, a public functional genomics dataset. The platform for GDS505 and GDS2881 was GPL96, [HG-U133A] Affymetrix Human Genome U133A Array, and for GDS507 and GDS2880 was GPL97, [HG-U133B] Affymetrix Human Genome U133B Array. The data consisted of 38 ccRCC tissues (9 in GDS505, 9 in GDS507, 10 in GDS2880, and 10 in GDS2881) and 36 matched normal tissues (8 in GDS505, 8 in GDS507, 10 in GDS2880, and 10 in GDS2881).

#### 2.1.2. Expression Analysis of DEGs

As described previously [10], all raw data of data normalization and gene differential expression analysis were performed with limma package (http://www.bioconductor.org/pack- ages/release/bioc/html/limma.html) in R. After the limma analysis, genes with |logFC| > 1 and* P* value (adj* P* value) <0.05 were deemed to be differentially expressed genes (DEGs).

#### 2.1.3. Gene Ontology and Pathway Enrichment Analysis of DEGs

The Database for Annotation, Visualization and Integrated Discovery [13] (DAVID, https://david-d.ncifcrf.gov, ver. 6.8) provides a comprehensive set of functional annotation tools for investigators to understand biological meaning. It was applied for pathway enrichment analysis (KEGG) with *P* value< 0.05 and Gene Ontology (GO) enrichment analysis with P value< 0.01 to analyze the DEGs.

#### 2.1.4. Protein-Protein Interaction (PPI) Network

With the confidence >0.4 and “Homo sapiens” as a limit, DEGs of PPI were gathered from String [14] (https://string-db.org, ver.10.5), a database to forecasted protein-protein interactions. The network visualization software Cytoscape was utilizing to generated PPI networks. Then, the top ten degree genes were chosen and deemed to hub genes using the plug-in unit: cytoHubba. The Oncomine Platform featuring scalability, high quality, consistency, and standardized analysis was utilized to investigate hub gene expression within ccRCC across multiple datasets.

#### 2.1.5. Survival Analysis

UALCAN [15], a user-friendly, interactive web resource for analyzing cancer transcriptome data, allows users to identify biomarkers and provides publication quality graphs and plots depicting gene expression and patient survival information based on gene expression. The overall survival analysis was constructed using UALCAN dataset.

### 2.2. Systematic Meta-Analysis

#### 2.2.1. Literature Search and Selection

Published reports on PubMed, Embase, Cochrane, Google Scholar, and CNKI were systematically investigated using the search terms “EGFR OR epidermal growth factor receptor” and “ccRCC OR clear cell renal cell carcinoma” to October 2018.

Studies which should satisfy the following conditions were screened by two investigators (Wang and Yu) independently. Discrepancies were resolved by a senior investigator (Chai).

Inclusion criteria were as follows: (1) all patients were diagnosed with ccRCC using cytological histopathology or cytology; (2) articles need to detect the alteration of EGFR expression in ccRCC by immunohistochemistry.

Exclusion criteria were as follows: (1) abstract, comment, review, and meeting; (2) EGFR expression detected by Western blot, RT-PCR; (3) lack of sufficient information; (4) duplication.

#### 2.2.2. Data Extraction

Two reviewers extracted data from eligible studies independently, including publishing information (first author, year, and journal), age, pathological grading, Furhman grading, Lymph node status, metastasis, and expression alteration.

#### 2.2.3. Quality Score Assessment and Publication Bias

As described by Zheng [16], Newcastle Ottawa Scale (NOS) was used to assess the quality of included trials. An asymmetry funnel plot was also used to evaluate the likelihood of publication bias in the meta-analysis.

#### 2.2.4. Statistical Analysis

All analyses were calculated with Review Manager software (Cochrane Collaboration, ver.5.3). Weighted mean difference (WMD) and 95% confidence interval (95% CI) were calculated by inverse variance (IV) method; for dichotomous data, Risk Ratio (RR) with 95% CI was calculated by Mantel-Haenszel (M-H) method. Cochran's *Q* statistic and Higgins' *I* squared statistic were used to assess the heterogeneity. When P value < 0.1 or I^2^> 50%, the heterogeneity was appeared and a random-effect model was applied, while P value  > 0.1 or I^2^< 50%, a fixed-model was applied [17, 18].

## 3. Results

### 3.1. Bioinformatics Analysis

#### 3.1.1. Identification of DEGs in ccRCC

**Figure 1:**
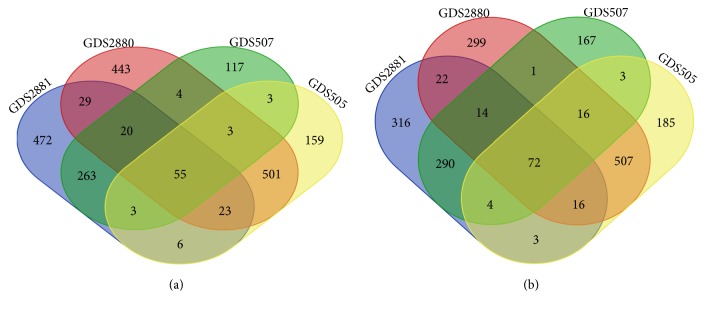
127 DEGs were identified in four profile datasets (GDS505, GDS507, GDS2880, and GDS2881). (a) 55 upregulated genes; (b) 72 downregulated genes.

**Figure 2:**
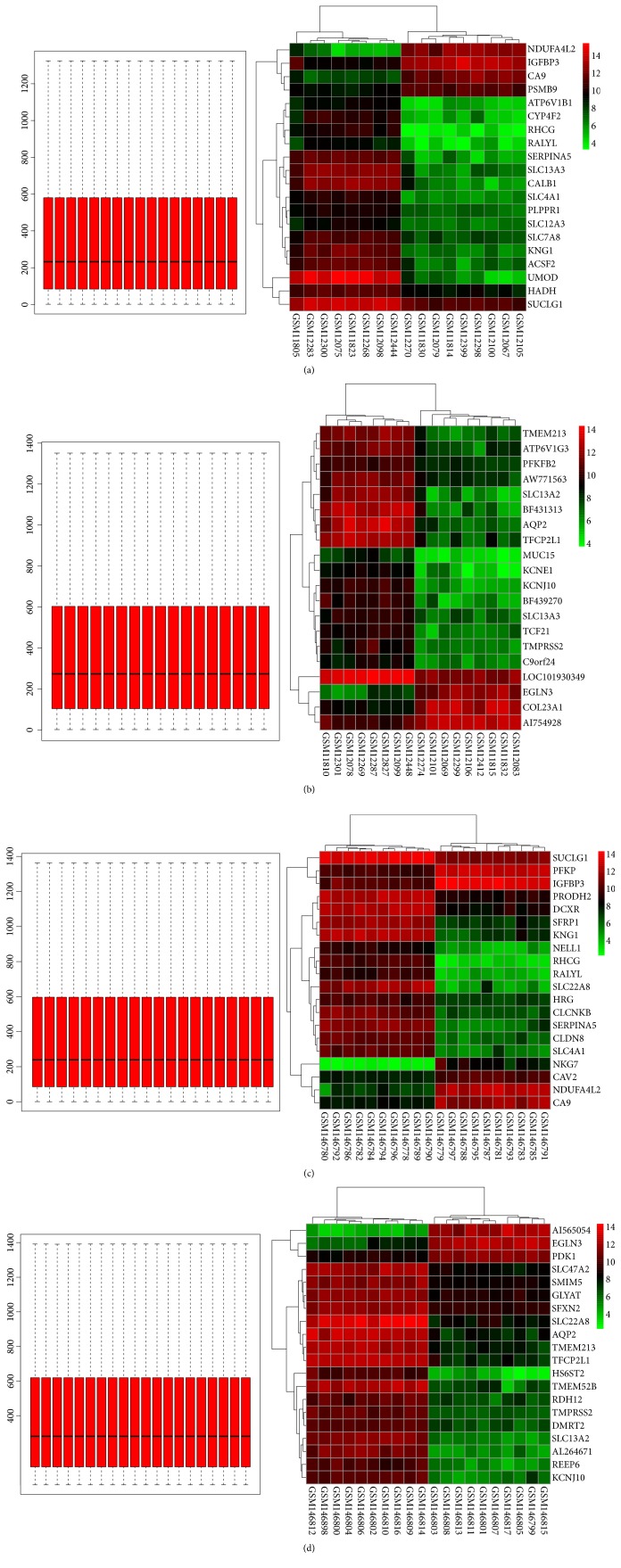
The normalization and cluster heatmaps of the top 20 DEGs in each dataset. (a) The normalization and cluster heatmaps of the top 20 DEGs in GDS505. (b) The normalization and cluster heatmaps of the top 20 DEGs in GDS507. (c) The normalization and cluster heatmaps of the top 20 DEGs in GDS2880. (d) The normalization and cluster heatmaps of the top 20 DEGs in GDS2881.

A total of overlapping 127 DEGs (Figure 1 and Table S1) were identified in four profile datasets (GDS505, GDS507, GDS2880, and GDS2881), including 55 upregulated genes and 72 downregulated genes (|logFC|>1 and* P* value<0.05). The cluster heatmaps of the top 20 DEGs and the results of normalization of each dataset were shown in Figure 2.

#### 3.1.2. GO and KEGG Analysis

**Figure 3:**
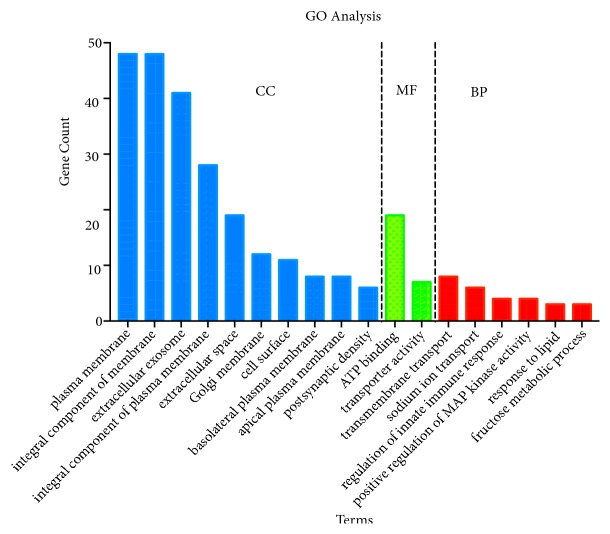
GO enrichment analysis of DEGs in clear cell renal cell carcinoma.

**Figure 4:**
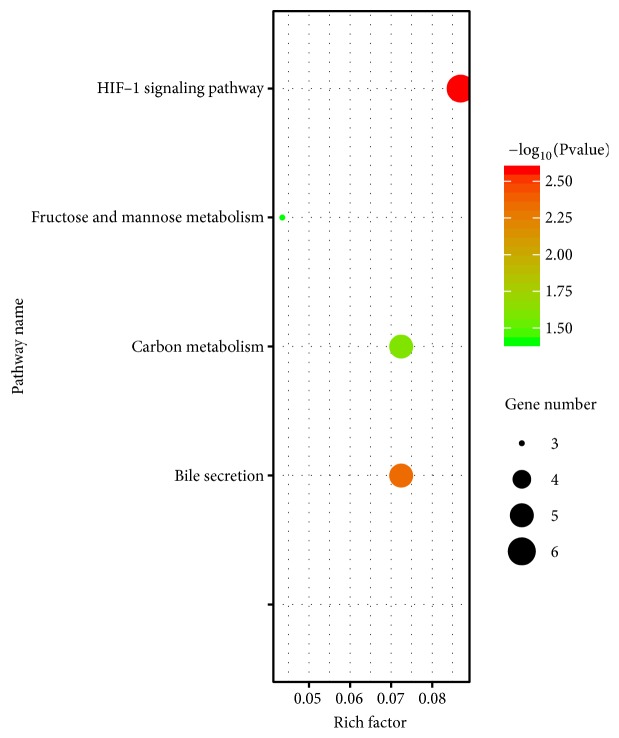
The pathways enriched for DEGs in clear cell renal cell carcinoma.

DAVID 6.8 was performed to analyze GO and KEGG analysis of DEGs in ccRCC. As shown in Figure 3, GO analysis consists of cell composition (CC) molecular function (MF) and biological processes (BP). GO analysis demonstrated that, in CC, DEGs of ccRCC were mainly enriched in 13 terms, such as integral component of membrane, extracellular exosome, and plasma membrane; in MF, DEGs were mainly enriched in 2 terms, ATP binding and transporter activity; in BP, DEGs were mainly enriched in 6 terms, such as transmembrane transport and sodium ion transport. Four pathways associated with DEGs were enriched (Figure 4), HIF-1 signaling pathway, bile secretion, carbon metabolism, and fructose and mannose metabolism.

#### 3.1.3. Protein-Protein Interaction (PPI) Network

**Figure 5:**
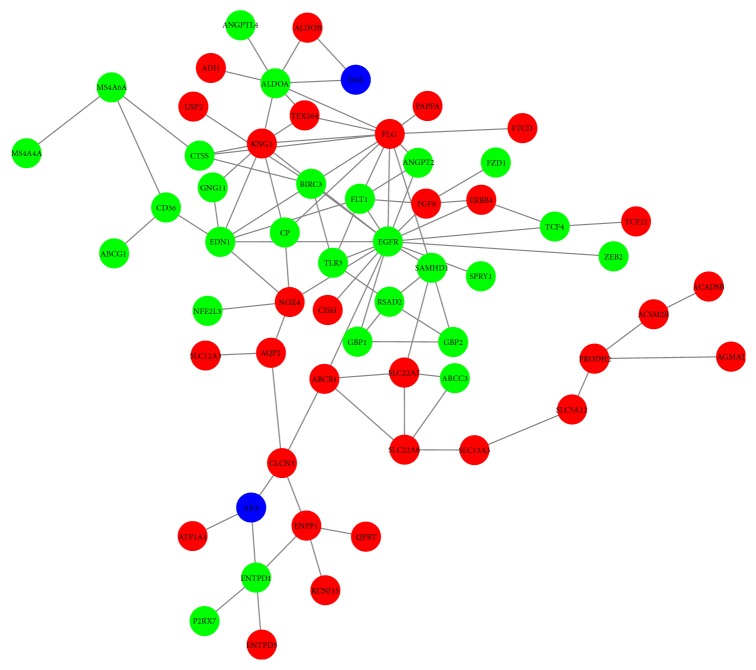
Protein-protein interaction (PPI) network (red nodes, green nodes, and violet nodes represent upregulated genes, down-regulated genes and other human proteins interacting with DEGs).

**Figure 6:**
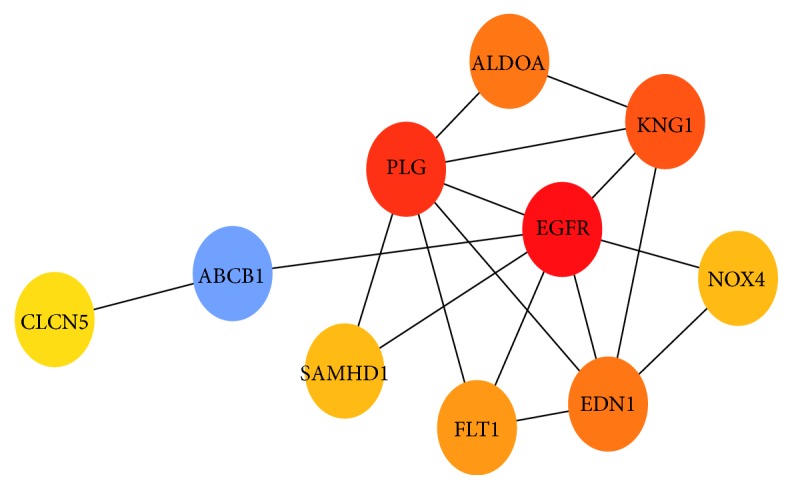
Ten top ten degree genes.

**Figure 7:**
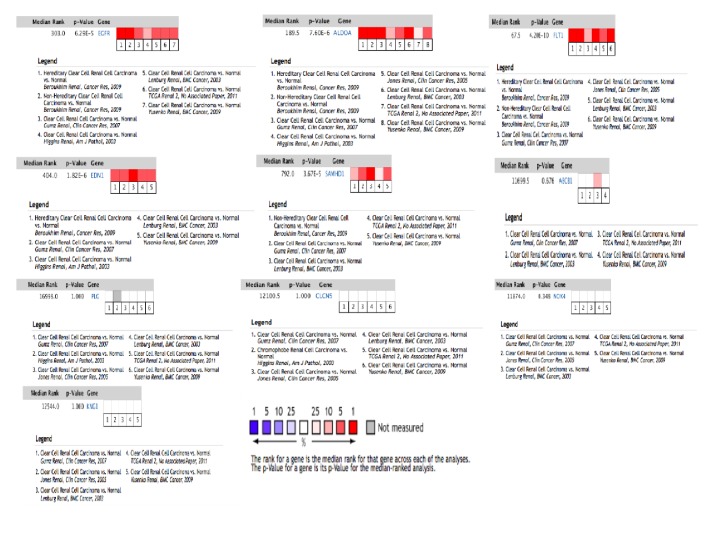
10 hub genes' expression among different analysis datasets.

We input DEGs into string to forecast protein-protein interactions, and then the date of PPI network was processed utilizing Cytoscape. In the PPI network (Figure 5), red nodes, green nodes, and violet nodes represent upregulated genes, downregulated genes, and other human proteins interacting with DEGs, separately. Using the plug-in unit, cytoHubba, ten hub genes were screened (Figure 6 and Table 1), including 5 upregulated genes (EGFR, EDN1, ALDOA, FLT1, and SAMHD1) and 5 downregulated genes (PLG, KNG1 NOX4, ABCB1, and CLCN5). We input hub genes into Oncomine Platform to investigate gene expression within ccRCC across multiple datasets. The results revealed that the expression of EGFR, ALDOA, PLT1, SAMHD1, and END1 in ccRCC had marked differences among different analysis datasets (Figure 7).

#### 3.1.4. Survival Analysis

**Figure 8:**
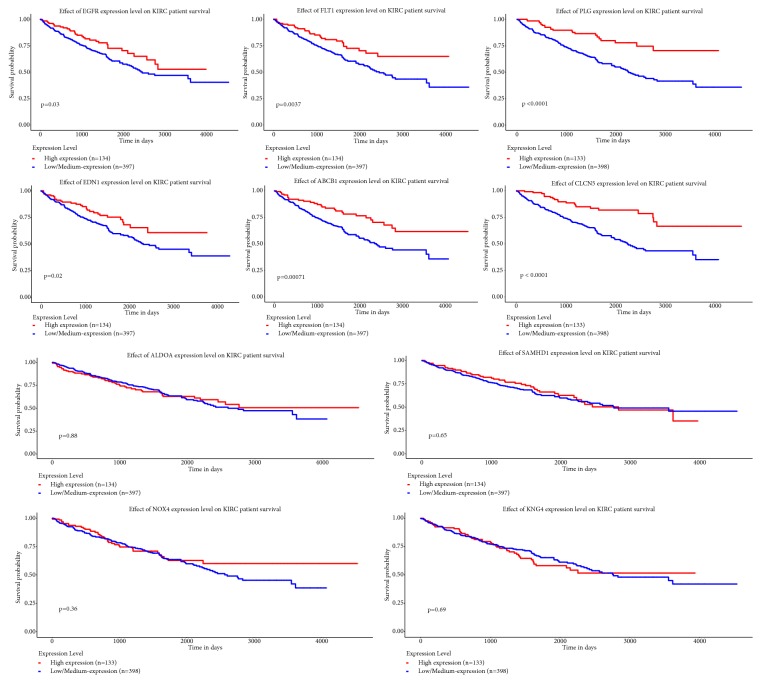
Prognostic values of 10 hub genes for overall survival in ccRCC patients. Patients were divided into low- and high-expression groups according to the median of each DEG expression.

The overall survival analysis of ten hub genes was performed by UALCAN dataset (Figure 8). The result revealed that high expression levels of EGFR, FLT1, PLG, EDN1, CLCN5, and ABCB1 were associated with worse survival of ccRCC patients.

### 3.2. Systematic Meta-Analysis

#### 3.2.1. Characteristics of Eligible Studies

**Figure 9:**
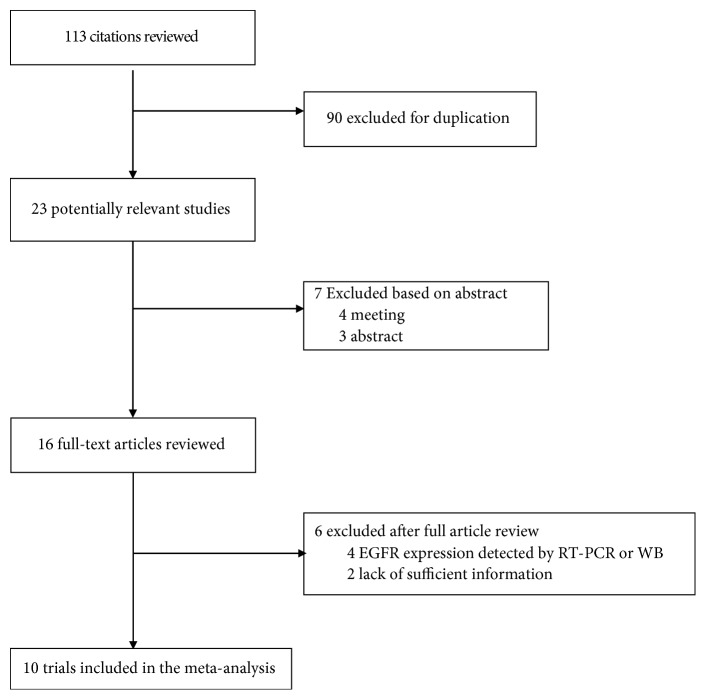
The study selection flowchart.

As shown in selection flowchart (Figure 9), a total of 113 published documents involving EGFR expression in ccRCC were identified with the literature search, and only 10 studies [19–28] met the inclusion criteria finally. Among the 10 eligible researches, 1513 ccRCC tissues and 88 normal tissues were involved in this meta-analysis. Five studies [21, 22, 24, 26, 28] were published in China, 3 studies [18, 19, 27] were published in Germany, 1 study [25] was published in Croatia, and 1 study [23] was published in Turkey, from 2005 to 2016. The baseline characteristics of 10 trials were summarized in Table 2.

In quality score assessment, 6 trials [20, 21, 24–26, 28] reached a score of 8, 2 trials [19, 23] reached a score of 9, 1 trial [27] reached a score of 10, and 1 trial [22] reached a score of 7.

#### 3.2.2. Association between EGFR Expression and ccRCC

**Figure 10:**
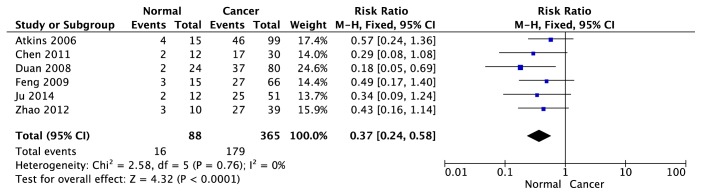
Forest plot for association between EGFR expression and ccRCC (cancer tissues versus normal tissues).

We analyzed the association between EGFR expression and ccRCC in 6 trials [20–22, 24, 26, 28] with 365 cancer tissues and 88 normal tissues. As indicated in Figure 10, there was no significant heterogeneity among individual trials (P=0.76; I^2^=0%). Accordingly, the statistical analysis would be carried out under fixed effect model. The combined effects demonstrated the expression of EGFR in ccRCC was higher than normal tissues (95% CI [0.24, 0.58], Z test = 4.32, p<0.0001). Funnel plot (Figure 15(a)) did not show the presence of publication bias.

#### 3.2.3. Association between EGFR Expression and Clinicopathological Parameters of ccRCC

**Figure 11:**
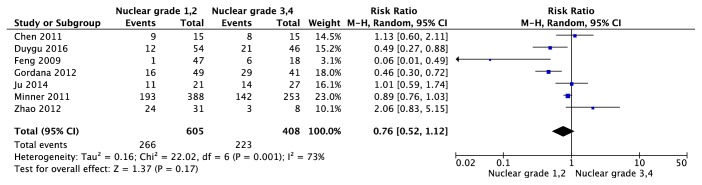
Forest plot for EGFR expression in ccRCC with different Furhman grading (Furhman grading 1,2 versus Furhman grading 3,4).

**Figure 12:**
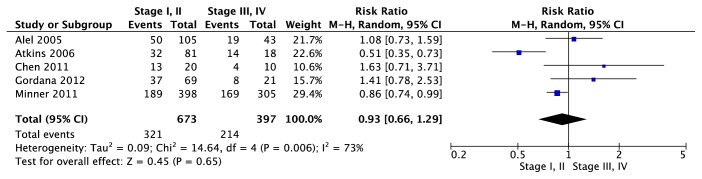
Forest plot for EGFR expression in ccRCC with different Pathological grading (T 1,2 versus T 3,4).

**Figure 13:**
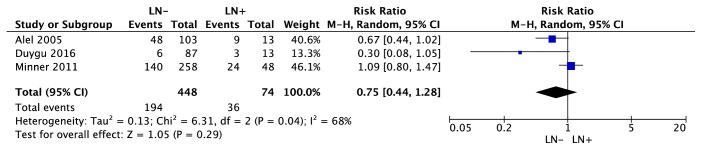
Forest plot for EGFR expression in ccRCC with different lymph node status (LN- versus LN +).

Association between EGFR expression and clinicopathological parameters was reported in 9 trials [19–21, 23–28]. Among 9 trials, 7 trials [21, 23–28] reported the association between EGFR expression and Furhman grading, 5 trials [19–21, 25, 27] reported the association between EGFR expression and Pathological grading, and 3 trials [19, 23, 27] reported the association between EGFR expression and lymph node status or metastasis. As indicated in Figure 11, using random-effects model (P=0.001; I^2^=73%), there was no difference in EGFR expression between Furhman grading 1,2 and Furhman grading 3,4 (95% CI [0.52, 1.12], Z test = 1.37, p=0.17). Also, in Figure 12, there was no difference in EGFR expression between T 1,2 and T 3,4 (95% CI [0.66, 1.29], Z test = 0.45, p=0.65) under the random-effects (P=0.006; I^2^=73%). For association between lymph node status and EGFR expression (Figure 13), meta-analysis showed that lymph node status was not correlated with EGFR expression (95% CI [0.44, 1.28], Z test = 1.05, p=0.29) under the random-effects model (P=0.04; I^2^=73%).

**Figure 14:**
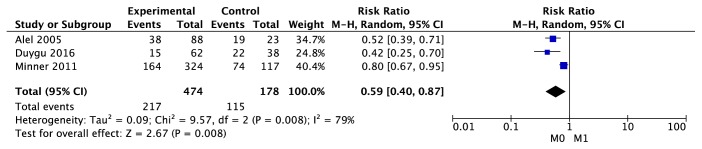
Forest plot for the association between EGFR expression and metastasis (M0 versus M1).

As indicated in Figure 14, there was significant heterogeneity (P=0.008; I^2^=79%) among the 3 trials reporting the association between EGFR expression and metastasis. The result showing a higher EGFR expression was detected in ccRCC with metastasis (95% CI [0.40, 0.87], Z test = 2.67, p=0.008).

**Figure 15:**
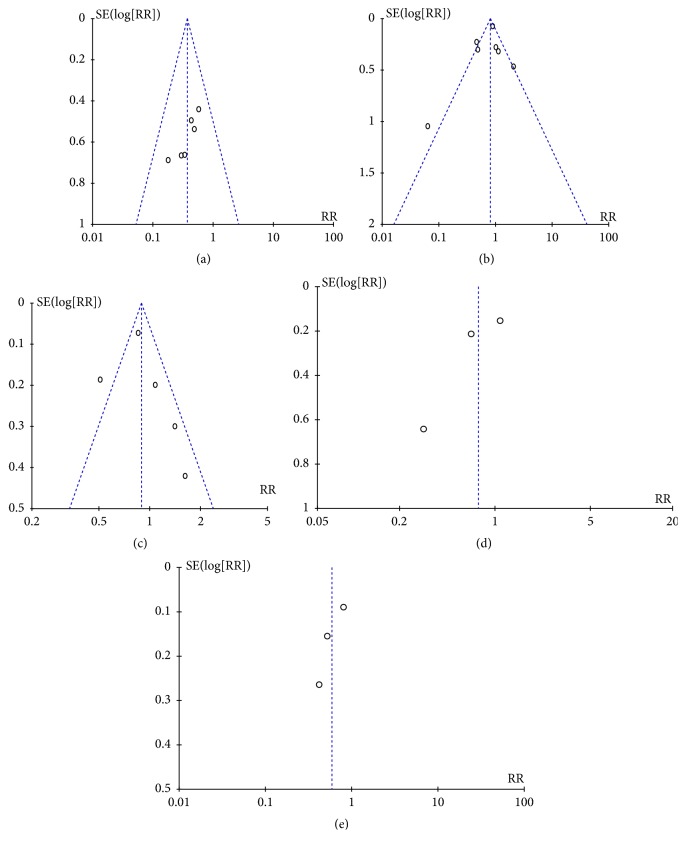
Funnel plot for publication bias test between EGFR expression and ccRCC or progression. (a) Funnel plot for association between EGFR expression and ccRCC (cancer tissues versus normal tissues). (b) Funnel plot for EGFR expression in ccRCC with different Furhman grading (Furhman grading 1,2 versus Furhman grading 3,4). (c) Funnel plot for EGFR expression in ccRCC with different Pathological grading (T 1,2 versus T 3,4). (d) Funnel plot for EGFR expression in ccRCC with different lymph node status (LN- versus LN +). (e) Funnel plot for Forest plot for the association between EGFR expression and metastasis (M0 versus M1).

The heterogeneity test of the association between EGFR expression and nuclear grade, pathological grading, lymph node status, or metastasis was performed as shown in Figures 15(b), 15(c), 15(d), and 15(e). The funnel plot showed that there was asymmetry in all 4 meta-analyses.

## 4. Discussion

ccRCC is one of the most common kidney malignancy [29] and accounts for ~3% of adult cancer [30]. Five-year survival of ccRCC patients with metastasis is only 12% and almost 20-40% patients would experience distant metastasis [5, 6]. It is important to understand the molecular mechanism of carcinogenesis and development of ccRCC. Microarray analysis with high-throughput sequencing technologies have been widely used to determine potential diagnosis and therapeutic targets in the progression of diseases [31, 32].

In the present study, a total of overlapping 127 DEGs (55 upregulated genes and 72 downregulated genes) were identified from four profile datasets. GO analysis revealed that 127 DEGs were mainly enriched in 21 terms, such as integral component of membrane, extracellular exosome, and plasma membrane. In addition, 127 DEGs were analyzed by KEGG analysis and showed that they were mainly enriched in 4 pathways. In the PPI network, ten genes with high degree were chosen as hub genes, including 5 upregulated genes (EGFR, EDN1, ALDOA, FLT1, and SAMHD1) and 5 downregulated genes (PLG, KNG1 NOX4, ABCB1, and CLCN5).

In order to further verify the relationship between ten hub genes and ccRCC, we compared the expression of hub genes across multiple datasets using the Oncomine Platform. Five genes (EGFR, ALDOA, PLT1, SAMHD1, and END1) had marked differences among different analysis datasets. Furthermore, overall survival analysis based on UALCAN revealed that high expression levels of EGFR, FLT1, PLG, EDN1, CLCN5, and ABCB1 were associated with worse survival of ccRCC patients.

Integrating the result from PPI network, Oncomine Platform, and survival analysis, EGFR, FLT1, and EDN1 were considered as key factors in the prognosis of ccRCC and potential targets for the treatment of ccRCC.

Epidermal growth factor receptor (EGFR), a member of receptor tyrosine kinases of the ErbB family, plays a significant role in promoting cell proliferation and opposing apoptosis [33–35]. Amplification and mutations of EGFR have been shown to be driving events in many cancers, like non-small cell lung cancer [36], renal carcinoma [37], and basal-like breast cancers [38]. For instance, Smith et al. [39] had identified that EGFR may be a critical determinant of HIF-2A-dependent tumorigenesis and a credible target for treatment of VHL / renal carcinoma.

**Figure 16:**
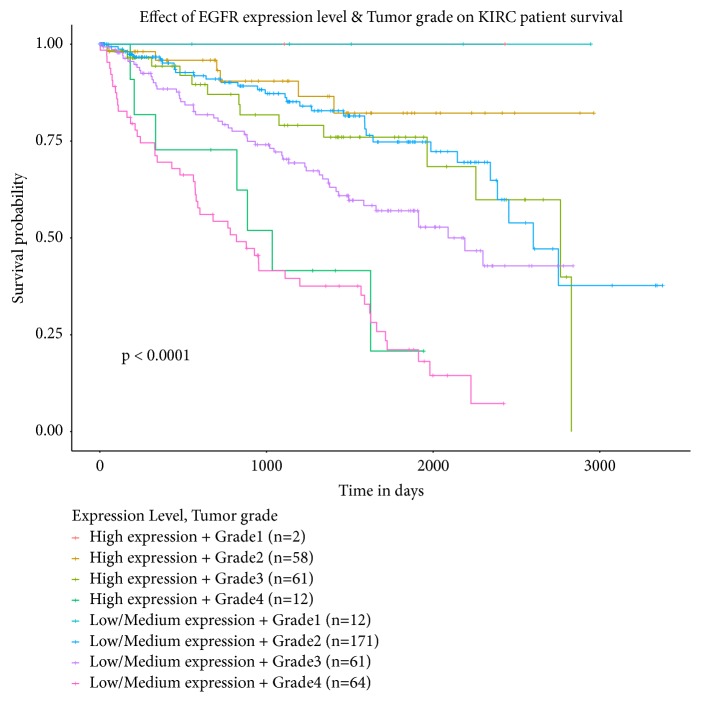
The association between EGFR expression and the survival rate of ccRCC with different pathological stage.

For better understanding the relationship between expression of EGFR and ccRCC, we performed a meta-analysis and explore the survival rate of ccRCC in different pathological stage using UALCAN dataset (Figure 16). To the best of our knowledge, this is the first meta-analysis to assess the association between EGFR expression and ccRCC. A total of 10 studies [19–28] were enrolled in this meta-analysis. The pooled results showed that there was a significant difference of EGFR expression between ccRCC tissues and normal tissues, and the expression of EGFR in patients with metastasis was higher. It was further testified that EGFR may be a potential biomarker and therapeutic target for ccRCC, especially patients with metastasis.

## 5. Conclusion

In summary, our study identified 127 DEGs, and 3 genes (EGFR, FLT1, EDN1) may be involved in the occurrence and progression of ccRCC based on integrated bioinformatic analysis. The results may contribute to a better understanding of ccRCC at the molecular level. Our meta-analysis showed that EGFR may be a potential prognostic biomarker and novel therapeutic target for ccRCC, especially patients with metastasis. However, further experimental studies both in vivo and in vitro are required to confirm the finding of this study, which may help to confirm identified gene functions and bring the mechanisms of ccRCC to light.

## Figures and Tables

**Table 1:** 10 hub genes (5 upregulated genes and 5 downregulated genes).

	Gene	Score		Gene	Score
	EGFR	17		PLG	12
up-regulated	EDN1	7	down-regulated	KNG1	8
genes	ALDOA	7	genes	NOX4	5
	FLT1	6		ABCB1	4
	SAMHD1	5		CLCN5	4

**Table 2:** The baseline characteristics of 10 trials.

Study	Country	Age	Case	Control	Outcome	Quality
Axel 2005	Germany	63	149	0	①③④	9
Atkins 2006	Germany	NA	99	15	①⑤	8
Chen 2011	China	56	30	12	①②⑤	8
Duan 2008	China	53.5	80	24	⑤	7
Duygu 2016	Turkey	58	100	0	②③④	9
Feng 2009	China	50.8	66	15	②⑤	8
Gordana 2012	Croatia	61	94	0	①②	8
Ju 2014	China	68.3	51	12	②⑤	8
Minner 2011	Germany	NA	711	0	①②③④	10
Zhao 2012	China	56.9	39	10	②⑤	8

NA: no mention; ① pathological grading, ② Furhman grading, ③ lymph node status, ④ metastasis, and ⑤ normal tissue.

## Data Availability

The data used to support the findings of this study are included within the article. The gene expression data can be accessed on Gene Expression Omnibus (GEO). The overall survival analysis of EDGs was acquired from UALCAN dataset.

## Supplementary Materials

Supplementary Materials In Table S1, a total of 127 DEGs (Figure 1 and Table S1) were shown, including 55 upregulated genes and 72 downregulated genes.
